# Effects of non-pharmacological or pharmacological interventions on cognition and brain plasticity of aging individuals

**DOI:** 10.3389/fnsys.2014.00153

**Published:** 2014-09-02

**Authors:** Valentina Pieramico, Roberto Esposito, Stefano Cesinaro, Valerio Frazzini, Stefano L. Sensi

**Affiliations:** ^1^Molecular Neurology Unit, Center of Excellence on Aging, University “G. d’Annunzio”Chieti-Pescara, Chieti, Italy; ^2^Department of Neuroscience, Imaging and Clinical Sciences, University “G. d’Annunzio”Chieti-Pescara, Chieti, Italy; ^3^Departments of Neurology and Pharmacology, Institute for Memory Impairments and Neurological Disorders, University of California-IrvineIrvine, CA, USA

**Keywords:** brain aging, neuronal plasticity, cognitive enhancing drugs, non-pharmacological interventions, cognitive enrichment

## Abstract

Brain aging and aging-related neurodegenerative disorders are major health challenges faced by modern societies. Brain aging is associated with cognitive and functional decline and represents the favourable background for the onset and development of dementia. Brain aging is associated with early and subtle anatomo-functional physiological changes that often precede the appearance of clinical signs of cognitive decline. Neuroimaging approaches unveiled the functional correlates of these alterations and helped in the identification of therapeutic targets that can be potentially useful in counteracting age-dependent cognitive decline. A growing body of evidence supports the notion that cognitive stimulation and aerobic training can preserve and enhance operational skills in elderly individuals as well as reduce the incidence of dementia. This review aims at providing an extensive and critical overview of the most recent data that support the efficacy of non-pharmacological and pharmacological interventions aimed at enhancing cognition and brain plasticity in healthy elderly individuals as well as delaying the cognitive decline associated with dementia.

## Introduction

Brain aging is associated with cognitive and functional decline and represents the favorable background for the onset and development of Alzheimer’s disease (AD). AD is becoming a major socio-economical challenge for society (Hurd et al., 2013). Early use of imaging and biological markers can identify at-risk for AD subjects decades before the appearance of cognitive decline. It is therefore imperative to act during this extended pre-clinical phase and promote endogenous responses in brains that still have a large cognitive reserve and optimal levels of neural plasticity. Beside pharmacological intervention, a great deal of interest resides on ways that allow modulation of brain plasticity in the elderly by acting on cognitive stimulation and/or aerobic exercise. Cognitive enrichment as well as physical activity can in fact promote neural plasticity, increase neurotrofism and reduce AD incidence. This review aims at providing an extensive and critical overview of the most recent data that support the efficacy of non-pharmacological and pharmacological interventions aimed at enhancing cognition in healthy elderly individuals. This line of intervention can be also critical in delaying the cognitive decline associated with dementia or other widespread neurodegenerative conditions like Parkinson’s disease (PD).

### Cognitive and neural reserve

The central nervous system (CNS) is a highly dynamic structure that undergoes continuous functional remodeling. Salient experience promotes widespread changes within neuronal circuits and brain areas, a process that results in the harmonization of cognitive skills that are set to meet and cope with demands of everyday life. In this context, CNS plasticity is the ability to adopt functional or structural responses (Ganguly and Poo, 2013) aimed at promoting successful adaptive behavior (Xerri, 2008; Wittenberg, 2010). Activity-dependent neural plasticity is not only required for optimal brain development but is also occurring through adulthood and senescence (Buonomano and Merzenich, 1998; Feldman and Brecht, 2005; Feldman, 2009). Plasticity requires a complex process of molecular, structural, and functional integrations that are carried out in neurons, glia, and subcellular compartments like dendrites, spines, and synapses. These biochemical and morphological changes go along with spatiotemporal synchronization of large neuronal populations across different brain regions. While neuronal plasticity has been studied mostly in the neocortex and hippocampus, it is important to underline that the phenomenon is common to almost all the CNS structures. The last two decades have produced a dramatic increase in the availability of techniques that allow the extensive investigation of CNS from its subcellular components to neural circuits.

Advancements in fluorescent microscopy, molecular biology, and electrophysiological techniques have helped to unravel many molecular determinants of neuronal plasticity. On the other hand, the availability of neuroimaging tools such as functional magnetic resonance imaging (fMRI), positron emission tomography (PET), and magnetoencephalography (MEG) have enhanced the capability to detect spatiotemporal patterns of brain activation that occur within networks, nodes, and neuronal pathways (Pascual-Leone et al., 2005; Raichle and Mintun, 2006; Grefkes and Ward, 2014). These technical advancements, along with the notion that the aging brain retains the capacity to reorganize its morphological and functional architecture, have promoted strong interest and leaps forward in the knowledge of the physiology of the aging brain and aging-related cognitive processes as well as in the exploration of strategies aimed at enhancing or maintaining cognitive skills in the elderly. This area of investigation is particularly relevant as molecular, structural and functional derangement of physiological aging represents the pathological pabulum for the onset and development of neurodegenerative processes that underlay conditions like dementia or PD.

Cognitive reserve is the concept that has been proposed to explain the functional adaptation occurring in conditions that are associated with brain damage, but where structural impairment does not translate in patent cognitive deficits. When discussing this process, a clear distinction should be made between brain reserve and cognitive reserve. Brain reserve refers to the amount of preserved CNS “hardware”, namely the integrity of synapses, dendrites, neurons, glia, and pathways while cognitive reserve indicates the CNS “software”, in short the brain capability to reorganize its activity by setting in motion compensatory cognitive mechanisms. Thus, the cognitive reserve concept imposes to shift the attention from brain morphological integrity to functioning (Stern, 2009).

Despite recent great progress occurring in the field, the relative contribution of cognitive and brain reserve to the maintaining of brain functioning throughout aging is not completely clear. The two processes interplay and it is well known that rehabilitative interventions can positively act on both phenomena (Voss et al., 2013).

Cognitive reserve is positively influenced by educational, environmental, nutritional as well as genetic factors (Mora, 2013). The modulation of cognitive reserve greatly influences the vulnerability to dementia and affects the clinical course of the disease. Cognitive reserve has been indicated as a resource to be has tapped on in AD patients who despite the presence of large amount of AD-related pathology show relative paucity of clinical symptoms and signs. The process is thought to require the formation of new synapses as well as the plastic remodelling and functioning of brain networks (Amieva et al., 2014).

The drawback is that these patients can go undetected for long time and then, when exhaustion of the cognitive reserve occurs, eventually show a precipitous cognitive decline (Amieva et al., 2014). This rapid progression is considered to be related to high pathological loads that were compensated by a high cognitive reserve that eventually have been subdued and collapsed (Wilson et al., 2004; Amieva et al., 2014).

Neuroimaging evidence support this idea and indicate that AD patients with greater cognitive reserve often show underlying greater AD-related pathology. In a subset of AD patients, PET measurements of resting regional cerebral blood flow (rCBF) have shown an inverse relationship between resting cerebral blood volume (rCBV) and years of education (Friedland et al., 1985; McGeer et al., 1990). These studies indicate that patients with high levels of education manifest more pronounced reductions in parieto-temporal rCBV when compared to less educated subjects presenting similar clinical features. Similar results were observed in studies evaluating protective effects of rehabilitative activities (Stern et al., 1995; Scarmeas et al., 2003). fMRI studies have revealed patterns of brain network activity that supports the concept of cognitive reserve (Stern et al., 2005). Neuroimaging studies revealed that elderly people engage additional brain regions to compensate for impaired functioning (Fjell et al., 2014). For instance, upon completion of a memory task, experimental data indicate that, compared to young people, elderly individuals show increased recruitment of the prefrontal cortex (PFC), a process that possibly compensates for the reduced activity of brain regions that support visual processing of information (Ansado et al., 2013). The phenomenon has been labelled as “age-related posterior-anterior shift”, or PASA (Davis et al., 2008). PASA seems to optimize cognition by inducing the inhibition of pre-potent responses (Corbetta et al., 2008; Vincent et al., 2008; Vallesi et al., 2011). Moreover, a recent study indicates that subjects performing attention tasks show an increased activity in bilateral PFC that positively correlates with enhanced performance (Davis et al., 2012).

## Non-pharmacological interventions

Age-related modulations and variations of cognitive and neural systems are dynamic and not irreversible processes. Physical or cognitive training can promote positive and lasting cognitive effects in elderly people and behavioral gains that may translate in more efficient procedural skills that help to cope with daily life activities. Non-pharmacological intervention can be divided in two approaches: aerobic training and cognitive stimulation (Jack, 2011; Vaughan et al., 2012).

### Aerobic training

Aerobic exercise is associated with increased preservation of cognitive functions (Colcombe et al., 2004). Exercise induces a cascade of molecular and sub/cellular processes that favor angiogenesis, neurogenesis, synaptogenesis as well as enhanced production of neurotrophins specially, the increased synthesis and release of brain-derived neurotrophic factor, BDNF (Deslandes et al., 2009; Coelho et al., 2013). The role of neurotrophins is crucial as decreases in BDNF levels are associated with age-related neuronal loss and BDNF reduction is also observed in patients affected by neurodegenerative or psychiatric disorders. Aerobic exercise elevates BDNF concentrations, increases hippocampal volumes (with the control group showing a reduction of volume of ~1.4% while the trained group showed a volume increase of ~2% over a 1-year period), and promotes better cognitive functioning in terms of executive functions, thereby suggesting that the trophin is a critical factor in mediating the protective and cognitive effects of this type of training (Erickson et al., 2013). Regular aerobic exercise improves executive function, attention, processing speed, memory, learning processes, and helps to preserve mental abilities throughout life span (Colcombe and Kramer, 2003; Curlik and Shors, 2013; Dresler et al., 2013). Exercise is also beneficial for subjects suffering from Mild Cognitive Impairment (MCI) or early-stage dementia (Smith et al., 2010).

Longitudinal studies suggest that engagement in exercise at young age leads to better cognitive performance upon senescence. The process seems to follow a dose- response effect as, in analogy with pharmacological intervention, more robust effects are obtained in individuals who in their youth have exercised more intensively (Middleton et al., 2010). Exercise appears to preferentially target selected brain regions and/or cognitive domains (Kramer et al., 2003; Colcombe et al., 2006; Erickson et al., 2013). Neuroimaging supports the idea of greater effects of exercise in the prefrontal and medial temporal cortices (Berchicci et al., 2013). The hippocampus appears to be particularly sensitive to exercise with trophic responses leading to increased hippocampal volume by ~2% in subjects undergoing training (Kerr et al., 2010; Erickson et al., 2011, 2013).

Overall, exercise-driven effects on specific cognitive domains like executive functions match morpho-functional changes in related brain regions (hippocampus, PFC, and basal ganglia) and neural networks. Elderly people who regularly exercise show brain volumes increases by ~2% in these critical areas while age-matched individuals who are less physically active undergo a 1.4% decrease in volumes (Erickson et al., 2010, 2011; Esposito et al., 2013).

### Cognitive training

Cognitive stimulation by employing interventions that make use of ecological or virtual environments is also effective to compensate age-related cognitive decline (Curlik and Shors, 2013; Park and Bischof, 2013). To date, several types of cognitive trainings are available. Several programs are aimed at improving memory (Richmond et al., 2011; Dresler et al., 2013), learning (Bailey et al., 2010), attention (Mozolic et al., 2011), executive functions (Basak et al., 2008), fluid intelligence (Jaeggi et al., 2008), mnemonic techniques, or global cognition (Klusmann et al., 2010).

Shedding some light on the molecular targets of cognitive stimulation, preclinical models have shown that enriched environments improve cognitive performances by inducing enhanced expression of genes encoding for neurotrophins or promoting synaptogenesis, dendrite formation and arborization (van Praag et al., 2000; Fratiglioni et al., 2004). The same mechanisms also occur in brains of elderly people (Mora, 2013).

Neuroimaging data show that memory-targeted training can induce positive fMRI changes as well as modification in cortical thickness of hippocampal subfields and increases in the volumetry of left hippocampal subregions like the CA3, CA4, and dentate gyrus (Engvig et al., 2012). Memory training also increases the thickness of the right fusiform and lateral orbitofrontal cortex, a phenomenon that has been positively correlated with enhanced memory performances (Engvig et al., 2010). Unfortunately, only few fMRI studies have unveil functional connectivity effects associated with cognitive intervention (Burhan et al., 2012; Engvig et al., 2014).

## PC-based training

Video games are not just teenager leisure material, they are gaining a place in the field of rehabilitation as tools to improve cognition in the elderly (Clark et al., 1987; Dustman et al., 1992; Goldstein et al., 1997; Basak et al., 2008). Results in this area of intervention are, however, often contradictory. Computer-based cognitive training for 6 months has been shown to stimulate multi-domain abilities like immediate memory and delayed memory or language (Miller et al., 2013). Game-based cognitive training, on the other hand, has shown no group effects on visuo-spatial navigational abilities and memory, though attention improvements have been found (Whitlock et al., 2012).

Interestingly, a recent study showed that computerized training targeted at amelioration of reaction times, inspection times, short-term memory for words, executive functions, visuo-spatial acuity, arithmetic competence, visuo-spatial memory, visual scanning, visual discrimination, and n-back working memory promoted no enhancement in these domains, but resulted in global improvement of processing speed (Simpson et al., 2012). The phenomenon is known as transfer effect. Moreover, evidence suggest that computer games designed to improve processing speed can enhance short-term memory and attention (Szelag and Skolimowska, 2012).

A very intriguing recent study has shown that cognitive capabilities can be significantly rejuvenated in the elderly (Anguera et al., 2013). Upon playing a video game targeted at increasing multitasking performance (a domain that show a physiological age-dependent decline), study subjects showed benefits not just in multitasking but also in untrained domains like sustained attention and working memory. The behavioral result was associated EEG evidence that show enhanced midline frontal theta power and frontal–posterior theta coherence in the trained group. More importantly, by simply undergoing a training period that spanned for just 12 h over a 4 week period, elderly subjects showed improved multitasking capability and matched performance levels of untrained young (20-year-old) subjects. Improved cognitive performances were maintained for 6 months after suspension of training, thereby suggesting long-lasting functional effects. Thus, the study supports the idea that age-related decline can be, not only halted, but actually reversed.

The field of PC-based cognitive training is still in its infancy, but, nevertheless, promising. Studies have started to analyze and debug areas that can potentially hamper the efficacy of this type of intervention. For instance, off the shelf, commercially available games appear to be less effective than personalized games (Peretz et al., 2011). Unfortunately, online games designed to enhance cognition in the elderly have generally failed mostly because of lack of motivation or ceiling effects in trainees who were already highly functioning and/or highly exposed to digital settings like virtual reality environments and games (Bozoki et al., 2013). Factors greatly affecting the cognitive outcome of PC-based trainings are the intensity and frequency of exposure to the training; however, a clear cut linear relationship between these factors and the cognitive output has, surprisingly, been difficult to be drawn (Whitlock et al., 2012; Wild-Wall et al., 2012). Better study designs are therefore needed (Green and Bavelier, 2008; Nouchi et al., 2012; Whitlock et al., 2012). Finally, in a recent comparative study, a custom-made computerized intervention was found to improve overall memory and attention performances in a group of elderly subjects. The study showed that training effects were still present 3 month after training withdraw (Zelinski et al., 2011).

## Combined training

Interest in multimodal interventions has been increased in recent years. Evidence, unfortunately still too few, suggest that strategies that combine aerobic training and cognitive stimulation have a significant synergistic value (Schneider and Yvon, 2013).

In a recent study, healthy elderly subjects were exposed to four different training conditions (Shatil, 2013). The first group was exposed to cognitive training, the second group engaged itself in moderate aerobic activity, the third group followed a combination of cognitive and aerobic training, while the control group was simply exposed to book-reading. Results of the study indicated that the third group, exposed to combined training, showed the best improvement in eye-hand coordination, working memory, long-term memory, and reaction times.

Interesting effects were also found in case of PC-based training combined with exercise. Cycling sessions performed in a virtual reality environment enhanced executive functions more effectively that regular cycling in a gym on stationary bikes (Anderson-Hanley et al., 2012). Positive effects of trained cybercyclists correlated with increase of BDNF. Important effects, in the cybercycling group, were also seen in MCI subjects. In this group, training reduced by 23% AD conversion (Anderson-Hanley et al., 2012). Another study (Maillot et al., 2012) found a significant improvement in executive control and processing speed after a 12 week aerobic training that employed the Nintendo Wii sports software console.

Other studies have supported the idea of the synergistic value of combining exercise and cognitive trainings to promote memory performance (Fabre et al., 2002; Oswald et al., 2006). Unfortunately, no data are available to verify whether these training-driven benefits translate in better performances in daily living activities. A previous study, from our group, has tried to fill the gap (Pieramico et al., 2012). We evaluated effects of 6-month combination training on cognition, functional connectivity and daily living activities. Thirty healthy elderly divided in two groups of 15 subjects were exposed to either no changes in their daily living routine (control group) or to a combination training consisting in exposure to cognitive, motor, and sensorial stimulations (trained group). Groups were evaluated, before and after the end of the training period, with neuropsychological and occupational tests, resting state fMRI and analysis of cortical thickness. Combination training improved cognitive and occupational performances, reorganized the functional connectivity of Default Mode (DMN) and Dorsal Attention (DAN) networks, and promoted lower cortical thinning in several brain regions (Pieramico et al., 2012). Interestingly, analysis of dopamine-related genes indicated that carriers of DRD3 ser9gly and catechol-O-methyltransferase (COMT) val158met polymorphisms had the greatest benefits from exposure to training, thereby suggesting a genetically-driven susceptibility to the positive effects of this type of combined intervention.

## Critical points in non-pharmacological interventions

Many unknowns are still present when dealing with training programs. For instance, the role played by intensity of intervention and knowledge on which is the most beneficial set of exercises to be employed are still largely unclear and missing. Also uncertain is what concerns the specificity and selectivity of factors that can promote significant, reproducible, and durable effects on cognition. Few studies have investigated correlations between frequency and duration of aerobic and cognitive trainings along with the overall morpho-functional changes and effects on brain functioning upon aging.

Furthermore, still not completely defined is for how long positive effects are maintained and therefore, robust well-designed longitudinal studies are needed.

A limiting factor that should be not overlooked is individual variability, an aspect that mandates more accurate customization of any given set of intervention. For instance, in analogy with the field of pharmacogenomics, a “cognitogenomic” approach, in which genetic differences are explored in relation to response to cognitive trainings, is definitely necessary (Pieramico et al., 2012).

## Pharmacological interventions

Pharmacological intervention aimed at enhancing or maintaining cognitive functions has been the focus of intensive research in recent years (Lanni et al., 2008; Hussain and Mehta, 2011; Lynch et al., 2011; Lynch and Mills, 2012; Wood et al., 2013; Urban and Gao, 2014). An intuitive definition of Cognitive Enhancer Drugs (CEDs) qualifies these molecules as able to enhance cognitive skills; however, an exhaustive definition of CEDs is, perhaps, not so simple. According to Lynch et coll., there is a need for a “dimensional” system to successfully define and classify CED candidates (Lynch and Gall, 2006; Lynch et al., 2011).

The first level, or dimension, is the target of action. Does the CED candidate mainly act on “general” psychological states like arousal or attentional level or does it work by potentiating single and/or selective cognitive domains? If the action is global, CED effects should be obtained through an optimization of the processing and information flux that operate within several brain nodes and networks. In case of selective activity, the drug should instead affect only specific and distinct subsets of cognitive domains.

Another important CED level/dimension is the neurobiological one or, in other words, the molecular mechanisms of action. Which neurotransmitter systems, receptor subpopulations, neuronal circuits, and/or brain structures are modified by CED activity?

Finally, a third dimensional level is represented by the distinction between drugs that enable to perform complex tasks in a more rapid or efficient way (efficiency augmentation) vs. molecules that promote enhanced performances (cognitive capability augmentation; Lynch, 2004; Lynch et al., 2011).

In accordance with this systematization, CEDs can be classified as drugs mainly acting on general or psychological aspects of cognition, compounds aimed at potentiating more specific cognitive domains or, at least in theory, drugs that are aiming at enhancing the global cognitive capability of healthy and already well performing individuals.

Most CED candidates are evaluated in preclinical models, sets where standardized behavioral outcomes are easily assessed. Although the translation of preclinical data in valid clinical outputs poses several limitations (see Sarter, 2006), animal models remain extremely valuable to identify CED neurobiology (Roesler, 2011).

In that respect, methylphenidate and modafinil, are good examples of a category of molecules that, across the board, induces changes of psychological variables such as vigilance, attention, and memory. Drug effects on arousal have been extensively described; however, a recent study that examined human subjects required to discriminate significant stimuli from distractors raised some question about the validity of the mechanism (Agay et al., 2010). Methylphenidate increases attention or “vigilance” by stimulating the cortical dopaminergic and noradrenergic transmission systems, a mechanism of action first described in preclinical settings and then confirmed by PET in human studies. Oral methylphenidate administration (0.25 mg/Kg) has been shown to promote a 50% blockage of the dopamine transporter (Volkow et al., 1998; Hannestad et al., 2010) and significantly increase extracellular dopamine concentrations. However, oral methylphenidate (at therapeutic ranges; 0.14 mg/kg) was also shown to bind with high affinity to the norepinephrine transporter. Methylphenidate-mediated effects on attention seem quite specific for tasks that need sustained vigilance; however, the drug appears to be less efficient when subjects are dealing with situations with levels of complexity that require selective attention (Advokat, 2010; Advokat et al., 2011). The compound can also improve spatial working memory in healthy individuals in conditions in which subjects are challenged with ex novo spatial problems (Elliott et al., 1997). Interestingly, experimental data confirmed drug-dependent improvements in spatial working memory. However, the study indicated that such benefits only occurred in subjects who were poorly performing at baseline, thereby suggesting that the drug can work only when significant amounts of cognitive reserve are available to be recruited (McGeer et al., 1990).

Modafinil is another example of drugs operating on vigilance or “attentional” states. The compound was first introduced as agent that counteracts excessive sleepiness associated with narcolepsy. Modafinil binds to forebrain dopamine transporters (Volkow et al., 2009; Zolkowska et al., 2009; Andersen et al., 2010) and promotes an increase in extracellular dopamine concentrations. The molecule can also bind to norepinephrine transporters in the human thalamus (Madras et al., 2006) and modulate orexinergic neurons in the hypothalamus (Ishizuka et al., 2010). Modafinil has been shown to promote marked increases in motor activity in mice (Simon et al., 1995; Stone et al., 2002), however, the effects are modest in rats and monkeys (Edgar and Seidel, 1997; Andersen et al., 2010). Finally, some studies have indicated drug-dependent improvement in sustained attention in modafinil-treated healthy subjects (Randall et al., 2005; Esposito et al., 2013).

An important bias to be considered when evaluating CEDs is represented by the fact that most studies, in animal models (Béracochéa et al., 2002) or humans (Turner et al., 2003; Baranski et al., 2004; Muller et al., 2004; Randall et al., 2005), are focused on effects on attention and working memory while other cognitive domains (such as long-term memory) are often neglected (Minzenberg and Carter, 2008). CEDs that selectively act on specific cognitive domains (memory and attention) belong to two classes of drugs that target the fast excitatory synaptic transmission responsible for communication within cortical networks. The first class acts on nicotinic receptors (alpha7 and alpha4 agonists) and modulates glutamate release while the second one, the ampakine family, facilitates fast glutamatergic transmission by acting on α-Amino-3-hydroxy-5-methyl-4-isoxazolepropionic acid receptors (AMPAR).

The cholinergic system is involved in several important aspects of cognitive functions including attention, learning, and memory. Classical CEDs enhance brain cholinergic tone by inhibiting acetylcolinecolinesterases (AChEIs), the enzymes responsible of acetylcholine degradation. AChEIs (rivastigmine, donepezil, galantamine) are approved for the treatment of AD patients; however, these compounds have failed in patients suffering from MCI (Raschetti et al., 2007), and very little data are available on cognitive effects on healthy adults. Rivastigmine has been shown to negatively affect episodic memory and improve motor learning and visuospatial functions in healthy elderly subjects (Wezenberg et al., 2005). Donepezil has produced mixed results, the drug improves cognitive performance in healthy young subjects (Yesavage et al., 2002) but has also been shown to cause attention and short-term memory deficits in healthy elderly subjects (Beglinger et al., 2005). Selective α4β2 nicotinic receptor agonists represent a new class of drugs acting on the cholinergic system and found to induce attentional and cognitive improvements in animal models (Howe et al., 2010). Agonists of α4β2 and α7 nicotinic receptors represent interesting examples of drugs that can potentiate the prefrontal cholinergic activity. These drugs promote an increase in fast glutamatergic transmission in the cortex, thereby enhancing attentional processes (Dunbar et al., 2007; Jiang and Role, 2008; Loughead et al., 2010). The proposed mechanism of action for α4β2 agonists is based on the hypothesis that the prefrontal cholinergic system plays a crucial role in the detection of novel and relevant stimuli, thereby increasing attentional performances (Bloem et al., 2014). The drug leads to amplitude augmentation of signal-evoked cholinergic transients when a relevant stimulus is administered to a subject.

Ampakines are positive allosteric AMPAR modulators that enhance fast excitatory transmission, promote induction and enhanced strength of long-term potentiation (LTP; Lynch, 2002). These compounds also increase the production of BDNF and related proteins (Lauterborn et al., 2000, 2003; Legutko et al., 2001; Lynch, 2002; O’Neill et al., 2004, 2005; Wezenberg et al., 2007). A recent study has described a role for the CX546 ampakine in promoting the proliferation and neuronal differentiation of stem/progenitor cells originating from the subventricular zone (Schitine et al., 2012), thereby underlying the role of these molecules in the differentiation of newborn neurons and in the shaping of neuronal circuits. The CX929 ampakine has been reported to promote LTP and counteract learning impairments in mice models of neurodevelopmental disorders. In one study, a 5 day treatment with CX929 in a mouse model of the Angelman syndrome, was reported to enhance fast EPSCs in hippocampal slices and increase long-term memory scores by 50% (Baudry et al., 2012). Ampakine are reported to improve performance in monkeys challenged with complex cognitive tasks (Porrino et al., 2005; Hampson et al., 2009) and brain imaging has demonstrated increased activity in the frontal and temporal cortices upon treatment (Porrino et al., 2005). The study revealed drug-induced activation of the precuneus, a cortical area usually less involved in task response (Porrino et al., 2005). This observation strongly suggests that ampakine administration may promote functional recruitment of additional brain areas, thereby modifying the neural substrates of cognition. Unfortunately, no ampakines are currently approved by FDA. The only molecule that reached clinical trials is CX-717. The compound was evaluated in Phase I for AD treatment but withdraw upon additional controversial results. Potential novel candidates are nevertheless being investigated for treatment of several neurologic and psychiatric disorders such as PD, schizophrenia, autism, and Attention Deficit Hyperactivity Disorder (ADHD).

Pharmaco-genomic approaches to be implemented for CED development and administration represent a promising new avenue in the field of cognitive augmentation. Traditionally these approaches have been mainly focused on the identification of genetic polymorphisms that can predict more effective drug responses in subjects already affected by neuropsychiatric disorders. However, in the past few years, studies have started to unravel the pharmacogenomic basis of individual response to CEDs occurring in healthy subjects. Factors to be considered are polymorphisms of genes regulating drug metabolizing enzymes, transporters, and receptors. These factors may profoundly influence the dose–response relationship to drugs (Roses, 2008). Differences in COMT genotypes (Val-Val vs. Met-Met) have been associated with variability in working memory performances and cognitive responses following amphetamine administration (Bilder et al., 2004). Apoliprotein E4 (apoE4), an allele of apolipoprotein E, and a risk factor for AD, is an interesting case. Quite paradoxically, ApoE polymorphism in young healthy carriers (i.e., subjects who have therefore a higher risk of developing AD later on in life) have the tendency to perform decision-making and prospective memory tasks better than, ApoE3 carriers (who are instead protected, by virtue of this genotype, from AD; Marchant et al., 2010).

## Critical points in pharmacological interventions

A critical point in the clinical output of CEDs is represented by the great variability of cognitive or behavioral responses of healthy individuals to these drugs. Several studies have begun to question whether such differences depend on genotypes and/or baseline levels of cognitive function (Kimberg et al., 1997; Mehta et al., 2000; Gibbs and D’Esposito, 2005; Frank and O’Reilly, 2006; Cools et al., 2007). For example, dopaminergic drugs have been shown to improve working memory only low performing subjects (Gibbs and D’Esposito, 2006). Methylphenidate or bromocriptine seem to improve working memory in low performers but impair performance in subjects with high baseline spans (Kimberg et al., 1997; Gibbs and D’Esposito, 2006). These contradictory results have been explained by the presence of an inverted U-shaped relationship between cognitive performance and dopamine receptor stimulation (Yerkes and Dodson, 1908). Indicating some potential neurobiological determinant of the phenomenon, dopamine synthesis has been shown to be lower in in the caudate nucleus of individuals with low working memory spans when compared to synthesis in subjects with high spans (Cools et al., 2008). Accordingly, subjects with low dopamine synthesis in the basal ganglia performed better in reversal learning tasks after bromocriptine administration when compared to treated subjects who were already showing high basal ganglia synthesis (Cools et al., 2009). Modafinil administration has also been shown to have most effective cognitive effects in low performing subjects at baseline (Randall et al., 2005; Finke et al., 2010; Esposito et al., 2013).

Baseline cognitive levels also affect the cholinergic modulation of cognition in healthy individuals. Donepezil increases cognition of healthy subjects whose performance declined after sleep deprivation but the drug induced cognitive impairments in subject without sleep loss (Chuah and Chee, 2008; Chuah et al., 2009).

Finally, it should be underlined that optimal brain functioning results from a tight control of synaptic function, circuits remodeling, and connectivity modulation. In response to extrinsic stimuli, neurons accordingly change the strength of their connections in order to avoid excessive excitation or inhibition (Pozo and Goda, 2010). Therefore, CED-driven excessive connectivity can be sometimes potentially problematic. In theory, increased excitatory synaptic transmission, along with reduced synaptic pruning, may induce unregulated synaptic plasticity and neuronal circuit overactivation, thereby promoting a low signal-to-noise ratio that actually may lead to impaired cognition (Belmonte and Yurgelun-Todd, 2003).

## Conclusion and future directions

In this review, we have summarized effects on cognition and brain plasticity of non-pharmacological and pharmacological interventions targeted on elderly individuals. The field is blooming but many issues remain unresolved. Areas that are in great need of advancement concern a better understanding of the role played by genes in the modulation of senescence and/or response to intervention. Disclosure of more detailed genetic and epigenetic roadmaps will help the design and implementation of tailored and personalized training programs. A more extensive implementation of “omics” (proteomics, lipidomics, metabolomics) approaches in combination with neuroimaging will also promote a better assessment of morpho-functional changes that are occurring in the trained brain as well as whole body.

Recent progress in digital technologies leads to foresee, in the near future, a more user-friendly implementation of trainings that can avoid personal computers and make instead use of smartphones, virtual or augmented reality portable devices. In that respect, the come of age of digitally-native generations will undoubtedly promote an extraordinary acceleration in that direction.

All these, pharmacological and non pharmacological, interventions can be envisioned to be effective not only in maintaining optimal levels of cognitive capabilities in healthy elderly individuals, but also as preventive measures to counteract the development of AD or PD as well as therapeutic tools that can be employed in the early stage of these neurodegenerative conditions.

## Conflict of interest statement

The authors declare that the research was conducted in the absence of any commercial or financial relationships that could be construed as a potential conflict of interest.
